# Alpha-Thalassemia in North Morocco: Prevalence and Molecular Spectrum

**DOI:** 10.1155/2019/2080352

**Published:** 2019-03-13

**Authors:** Achraf Laghmich, Fatima Zahra Alaoui Ismaili, Amina Barakat, Naima Ghailani Nourouti, Mohamed Khattab, Mohcine Bennani Mechita

**Affiliations:** ^1^Biomedical Genomics and Oncogenetics Research Laboratory, Faculty of Sciences and Techniques of Tangier, University Abdelmalek Essaâdi, Tangier 90000, Morocco; ^2^Pediatric Hemato-Oncology Service, Children's Hospital, CHU Rabat 10010, Morocco

## Abstract

Unlike the other hemoglobinopathies, few researches have been published concerning *α*-thalassemia in Morocco. The epidemiological features and the mutation spectrum of this disease are still unknown. This regional newborn screening is the first to study *α*-thalassemia in the north of Morocco. During the period from January 2015 to December 2016, 1658 newborns umbilical blood samples were investigated. Suspected newborns were screened for *α*-globin defects using Gap-PCR and Multiplex Ligation-dependent Probe Amplification technique. The prevalence of *α*-thalassemia, its mutation spectrum, and its allelic frequencies were described for the first time in Morocco. Six different *α*-globin genetic disorders were detected in 16 neonates. This screening valued the prevalence of *α*-thalassemia in the studied population at 0.96% and showed the wide mutation spectrum and the heterogeneous geographical distribution of the disease. A high rate of carriers was observed in Laouamra, a rural commune in Larache province. Heterogeneity of *α*-globin alleles in Morocco explains the high variability of *α*-thalassemia severity. This diversity reflects the anthropological history of the country. These results would contribute to the prevention of thalassemia in Morocco directing the design of a nationwide screening strategy and awareness campaign.

## 1. Introduction

*α*-Thalassemia represents a group of recessively inherited hemoglobin disorders due to deficient or absent synthesis of *α*-globin genes. Affecting 5% of the world's population, *α*-thalassemia is the most common monogenic disorder [1]. The clinical phenotype of *α*-thalassemia varies according to the number of affected genes from almost asymptomatic to a lethal hemolytic anemia. More than 80 different genetic defects have been described; most of them are deletions and less commonly nondeletional defects involving *α*-globin genes [2, 3]. This leads to the wide spectrum of hematological and clinical phenotypes. Genotype-phenotype correlation is still not well documented [4].

*α*-Thalassemia is prevalent in populations of Southeast Asia, Central Africa, and the Middle East [5, 6]. In the last few decades, massive population migrations favored the spread of these defects. Some abnormal Hb variants, once specific to particular ethnic groups, are now worldwide spread [7, 8]. Morocco, a Mediterranean country, is a potential predilection zone of hemoglobinopathies due to its geographical position. The frequency of the *α*-mutated genes is believed to be increased due to the country's socioeconomic and cultural system encouraging consanguinity, the selective pressure of malaria in endemic close countries, and its history as a migration crossroad between sub-Saharan Africa and Europe [9, 10]. The World Health Organization estimates the rate of hemoglobinopathies carriers in Morocco at 6.5%, with more than 30 000 cases of major forms [11]. A previous regional study highlighted the region of North Morocco as a geographical hot-spot for hemoglobinopathies due to its high consanguinity rate already estimated and its location in the malaria belt [10, 12]. Nevertheless, very scarce data about the occurrence and epidemiology of *α*-thalassemia is available. Thus, the overarching aim of our present study is assessing the prevalence and spectrum of *α*-thalassemia in the north of Morocco. Our conclusions would help decoding the context of the disease epidemiology and lead to a better formulation of country-specific and optimized *α*-thalassemia policies.

## 2. Population and Methods

**Figure 1:**
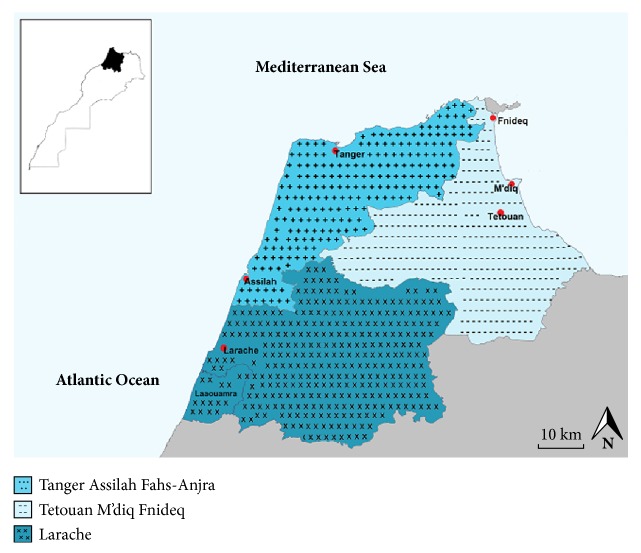
Geographic location of the region concerned by the study.

A total of 1658 newborns were screened for *α*-thalassemia in the three provincial hospitals of Tangier, Tetouan, and Larache cities between January 2015 and December 2016. The studied region is located in the northwestern part of Morocco and on the southern Mediterranean coast of the thalassemic belt (Area: 7098.8 km2, Population: 2386852 inhabitants (national census of 1st September 2014) [13]). The choice was based on the region's history with malaria, its high consanguinity, and the increase in length of stay and cumulative hospitalized days due to hemoglobinopathies in the region hospitals.  Figure 1 shows the geographic location of the studied region divided into three provinces: Tangier Assilah Fahs-Anjra, Tetouan M'diq-Fnideq, and Larache (Figure 1).

Our study is a newborn screening, using new techniques of molecular biology, for an accurate determination of the *α*-thalassemic genotypes and their frequencies. This can lead diagnostic centers to develop an effective genetic counseling of couples and a better diagnosis.

Umbilical blood samples of 4ml volume were taken by qualified midwives immediately after delivery on EDTA tubes from newborns in the hospitals. Each sample was accompanied by prior consent signed or thumb printed by the mothers. All the parents were of the region descents and were well informed about the study procedures and objectives. Information sheets with nationality, sex, age, dialect, and natives or not and written consent forms were available in Arabic to ensure comprehensive understanding of the study objectives. This study was approved by the Ethics Committee for Biomedical Research in the Faculty of Medicine and Pharmacy of Rabat (CERB), Morocco.

Several Researches have studied the appropriate cutoff values of hematologic features for *α*-thalassemia. In adults, *α*-thalassemia carriers' diagnosis is based on microcytosis and normal HbA2 level [14]. In newborns, an effective primary screening with 100% accuracy could be carried out using mean corpuscular volume less than 95fL, mean corpuscular hemoglobin less than 30 pg, and/or the presence of hemoglobin Bart's fraction [15, 16]. These were the inclusion criteria in our study.

All the newborns were analyzed for hematological indices, including red blood cells (RBC), hemoglobin concentration (Hb), mean cell volume (MCV), mean cell hemoglobin (MCH), and mean corpuscular hemoglobin concentration (MCHC) using an automated cell counter SYSMEX XT-1800 (Kobe, Japan). Ferritin level was assessed with established protocols using the automated VIDAS-BIOMERIEUX Immunoanalyzer, and the Hb separation studies were done by capillary electrophoresis method (MINICAP Sebia, Paris, France).

Genomic DNA was isolated from leukocytes using the QIAamp® Blood Mini Kit (Qiagen, Germany) and stored at − 20°C. Molecular screening of most common *α*-globin mutations (-*α*^3.7^, -*α*^4.2^) was performed using Gap-PCR (Gap-Polymerase Chain Reaction) as previously described by Dodé and Samuel S. Chong [17]. MLPA analysis was carried out using the SALSA MLPA Kit, HBA140-B4 Kit (MRC- Holland, Netherlands), according to the manufacturer's instructions [18]. It was used to detect copy number changes of 26 different sequences in the *α*-globin cluster on chromosome 16p13.3. PCR was performed with a standard thermocycler (Eppendorf, Germany), and the separation of the amplification products was done by capillary electrophoresis on the Genetic Analyser 3130XL (Applied Biosystems®). Then the results were analyzed with Coffalyser® (Applied Biosystems®). The deletional origin of *α*-thalassemia was investigated as no point mutation was detected in the studied region during routine diagnosis tests. Deletions are known to be responsible for more than 95% of *α*-thalassemia defects in the world [19]. This was performed in Biochemistry and Molecular genetics laboratory at Cadiz University, Spain.

Our data were analyzed using SPSS version 11.5 statistical software. An independent-sample test was carried out to compare the different hematological parameters of normal subjects and those suspected of being *α*-thalassemic. Statistical significance was assessed as a test with p ≤ 0.05.

## 3. Results

Our study estimates the prevalence of *α*-thalassemia, its mutations spectrum, allelic frequencies, and its geographical distribution in the north of Morocco. The hematological analysis revealed 26.59% of anemic newborns (441/1658). The Complete Blood Count profile was similar in the three provinces with a mild increase of hypochromic anemia in Larache province (Table 1).

Microcytosis was always accompanied by Hypochromia and it was found in 264 newborns. Hypochromic nonmicrocytic anemia was assessed at 67 newborns. The assessed iron status revealed a heterogeneity in the distribution of ID in the three studied provinces. Significant increase of low ferritin was observed in Tetouan M'diq-Fnideq province, while newborns from Larache province registered significant higher levels of cord blood ferritin. The ID was found in 173 newborns in the studied region (10.43%).

Capillary electrophoresis of hemoglobin was assessed to quantify the different Hb variants in the newborns. No newborn has shown Hb H, Hb C, Hb E, or Hb D variants. Hb Bart's was identified in 17 newborns, 2 in Tangier Assilah Fahs-Anjra province, 2 in Tetouan M'diq-Fnideq province, and 13 in Larache province. The percentage of Hb Bart's carriers at birth was estimated at 1.02% (17/1658). The highest frequency was registered in Larache province with 2.39% (13/542).

**Figure 2:**
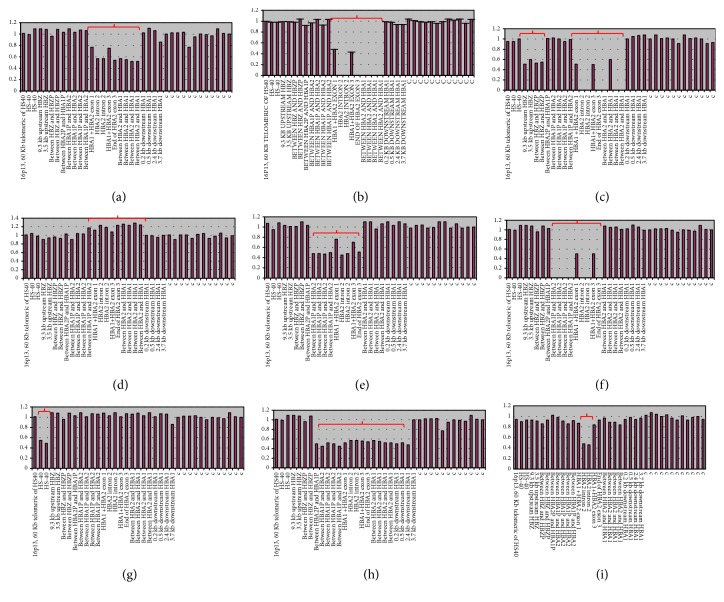
Multiplex Ligation-dependent Probe Amplification results. (a) Heterozygous deletion *α*^3.7^; (b) homozygous deletion *α*^3.7^; (c) homozygous deletion *α*^3.7^ with *ζ* deletion in cis; (d) *ααα*anti ^3.7^ deletion; (e) heterozygous deletion *α*^4.2^; (f) homozygous deletion *α*^4.2^; (g) HS-40 deletion; (h) -* *-^MED-I^  deletion; (i) African polymorphism.

Gap-PCR technique detected the presence of 11 *α*-thalassemic cases, resulting from two different molecular defects: -*α*^3.7^ deletion and –*α*^4.2^ deletion. The results of MLPA confirmed the cases diagnosed by Gap-PCR and revealed new *α*-thalassemic cases with novel mutations detected for the first time in the Moroccan population. Six different molecular defects on the-*α* globin gene are detected defining 8 genotypes (Table 2). MLPA results also showed a newborn with the African polymorphism *α*2. This polymorphism, detected for the first time in the Moroccan population, does not lead to thalassemia (Tables 2 and 3 & Figure 2). All the detected newborns carrying *α*-thalassemic defects were anemic at birth.

A heterogeneous geographical distribution of *α*-thalassemia in the studied region was revealed. The highest frequency was registered in Larache province 2.02%. However, Tangier Assilah Fahs-Anjra province and Tetouan M'diq-Fnideq province registered significantly low frequencies 4.73x10^−3^% and 4.14 x10^−3^%, respectively. *α*-Thalassemia prevalence was valued at 0.96% (16/1658) in the studied region. A preponderance of males was recorded with a frequency of 56.25% against 43.75% for females with a male to female sex ratio of 1.28.

-*α*^20.5^, -* *-^FIL^ , -* *-^THAI^ , -* *-^SEA^ , -* *-^Dutch1^  deletions were not found in any studied newborn. Among the 16 newborns who had *α*-thalassemia molecular defects, only 12 carried Hb Bart's at birth.

Comparison of the hematological and electrophoretic data of newborns with the same mutation and between newborns with different mutations has shown a significant difference in phenotype severity between the silent carries and the alpha-thalassemic newborns. -* *-^MED-I^  deletion and HS-40 deletion were associated with the most severe phenotypes.

## 4. Discussion

Hematological studies showed that 26.59% of studied newborns were anemic; 59.86% of them had microcytic hypochromic anemia (MHA), considered as the hallmark feature of *α*-thalassemia [20]. To our knowledge, no neonatal anemia study was reported in Morocco. The obtained anemia rates are similar to other studies in adults in Morocco [21, 22]. Hb Bart's carriers in the population were estimated at 1.02%. This rate is the lowest reported in Africa (7.38% in Tunisia [23], 10% in Algeria [24], 14.5% in Congo [25], and 11.5% in Tanzania [26]). Three quarter (12 neonates) of the newborns with Hb Bart's at birth had *α*-thalassemia molecular defects. Our results are similar to those reported in the Tunisian population [23].The absence of Hb Bart's in cord blood does not exclude the silent carrier state and a mild increase of Hb Bart's fraction does not ascertain a loss of *α*-globin loci [27, 28]. Four diagnosed *α*-thalassemic newborns showed no Hb Bart's fraction (Table 2). Five nonthalassemic newborns showed a fraction of Hb Bart's (not microcytic not hypochromic). Four of them registered a fraction of 0.1% each and one showed a fraction of 0.3%. The cutoff was estimated already at 0.2% by Munkongdee T et al. [29]. The healthy newborn exceeding the already estimated cutoff was investigated for point mutations without positive result. In our samples the cutoff was estimated at 0.3%; more studies should be done to confirm this result.

*α*-Thalassemic male to female sex ratio was estimated at 1.28. This is in agreement with other studies carried out in other populations (Bangladesh 1.26 [30], India 1.14 [31], Iran 1.21 [32], Tunisia 1.24 [33], Lebanon 1.32 [34]). This variance is noteworthy and merits further study, as thalassemia is a single gene disease transmitted by a recessive mode on inheritance. No difference in the prevalence of thalassemia between both sexes has been reported [35, 36].

No hydrops fetalis syndrome was diagnosed during the study. The rare hemoglobin variants already described in the Moroccan, Tunisian, and Algerian population, Hb H, Hb C, Hb O-Arab, Hb D-Punjab, Hb Philadelphia, and Hb Lepore, were absent in the region [25, 26]. This absence is evidence for their low prevalence in the studied region. Unpublished archives in the medical centers describe some rare cases of Hb O-Arab and Hb C variants, and hence the need for an enlarged sample's number screening for a precise determination of these variants.

Genetic analysis identified six different *α*-thalassemia defects, diagnosed for the first time in the Moroccan population. In Tunisia, nine mutations were described to be responsible for this disease [37], and six in the Algerian population [38]. The regional prevalence being estimated at 0.96% seems to be the lowest in the Mediterranean basin (Algeria 4.6%, Tunisia 4.8-5.4%, Libya 1-5%, Cyprus 10.6%) [39, 40]. Larache province was the most affected with a frequency of 2.02% (11/542). This disproportioned distribution between the three provinces could be explained by the different consanguinity rates, different history with malaria, and disparity in terms of the availability of primary care services and genetic counseling [11].

-*α*^3.7^ deletion is the most common mutation found in the North Moroccan population (allele frequency 0.33%). However, it is less frequent compared with the other Mediterranean countries (Algeria 2.9% [38], Tunisia 4.5%, Sicily 4.1% in 2002 [37], and Cyprus 7.7% in 2000 [39]). -*α*^3.7^ deletion was associated, in one newborn, with a heterogeneous deletion in cis on the *ζ* gene already detected in his father. To our knowledge, this association was never reported before in literature.

In our study -*α*^4.2^ allele was found at 0.12% in our samples. Higher frequencies were registered in Bahrein 2% [41], Tunisia 0.9% [37], and Malaysia 0.6% [42]. −*α*^anti3.7^ was reported in our samples at 0.06%. It was estimated at 1% in the Cyprus population in 2000 [39]. HS-40 deletion allele and *α*0 -* *-Med-I allele were found in lower frequencies (0.03% each). HS-40 deletion, already reported in the Brazilian population [43] but never in North Africa and Middle East [38, 44], is caused by the loss of the major regulatory element of the human alpha-globin locus, located 40 kb upstream of the zeta-globin gene, essential for *α* globin expression. -* *- MED-I, also responsible for *α*0-thalassaemia, is relatively frequent in Greece, Turkey, and the Middle East [3]. In Algeria this mutation was reported at 0.3% [38], Cyprus at 0.5% [39]. MLPA results showed also the existence of the African polymorphism with a frequency of 0.03%; this polymorphism is not related to any thalassemic phenotype. In Tunisia this polymorphism has an allele frequency esteemed at 0.99% [37].

-*α*^3.7^, -*α*^4.2^, and -* *-^MED1^  were already reported together in North Africa and Arab world [38, 45, 46]. This suggests that the origin of the common mutations spread is the same because of the ongoing genetic exchange all along this region over time. The possible introduction of these mutations in North Africa by Arab conquests was already discussed [41, 47, 48].

The eight found genotypes are responsible for intermediate phenotypes varying between the silent carrier and the classical trait due to the loss of one or two *α*- loci per chromosome. The rarity of hydrops fetalis and Hb-H disease in the studied population can be explained by the infrequency of the loss of three and four *α*- globin loci. Our work confirms previous observations describing the North African population as extremely heterogeneous [49]. The differences between the Moroccan and the Algerian and Tunisian *α*- thalassemia molecular spectrum are very notable and reflect the historical background differences of each country.

## 5. Conclusion

Our results have enriched the mutation spectrum of thalassemia in the north of Morocco, which has provided necessary information for its diagnosis. Carriers' detection, genetic counseling, prenatal diagnosis, and focused prevention programs usually lead to a fall in births of affected newborns. Thus, we highly recommend that people in Larache province with microcytic hypochromic anemia strongly benefit from a routine thalassemia screening.

## Figures and Tables

**Table 1:** Complete Blood Count and ferritin profile in the north of Morocco.

Province	population	Anemic	Non Anemic	Microcytic hypochromic anemia	Other anemia*∗*	Ferritin level		
						Low	Normal	High
Tangier, Assilah-Fahs-Anjra	633	163	470	79	84	52	11	16

Tetouan, M'diq, Fnideq	483	109	374	66	43	46	5	15

Larache	542	169	373	119	50	75	9	35

*∗* means other anemia: macrocytic normochromic, macrocytic hypochromic, normocytic normochromic, and normocytic hypochromic.

**Table 2:** Mutations and their allelic frequencies in the studied region.

Molecular defect	Allele frequencies (%)	Genotypes	N
-*α*^3.7^	0.33	-*α*^3.7^/ *αα*	6
		-*α*^3.7; *ζ* del^ / *αα*	1
		-*α*^3.7^/ -*α*^3.7^	2

-*α*^4.2^	0.12	-*α*^4.2^/ *αα*	2
		-*α*^4.2^/ -*α*^4.2^	1

-*ααα*^anti 3.7^	0.06	-*ααα*^anti 3.7^/ *αα*	2

HS-40	0.03	*αα*/ *αα* with HS-40 del	1

-* *-Med I	0.03	-* *-^Med I^ /*αα*	1

**Table 3:** Hematologic features and hemoglobin Bart's levels by genotype.

Genotype	Province			Hb (g/dl)	MCV (fl)	MCH (pg)	Hb Bart's %
	Tangier Assilah Fahs-Anjra	Tetouan M'diq Fnideq	Larache				
-*α*^3.7^/ *αα*	2	1	3	11.5	126.2	35.4	0.8
				13.1	92.6	29.2	0
				13.5	94.7	29.9	1.8
				12.5	92.5	28.7	0.45
				15.5	93.5	29.6	0.9
				12.5	94	33.8	0

-*α*^3 .7^/-*α*^3.7^	0	0	2	11.1	95.9	29.9	1.8
				13.3	98.5	31.5	2.3

-*α*^3.7; *ζ* del^ /*αα*	0	0	1	11.8	96.3	30.3	1.3

*ααα* ^anti 3.7^/*αα*	1	0	1	11.2	93.3	28.5	0
				11.4	93.7	28.9	0

-*α*^4.2^/ *αα*	0	0	2	11.9	95.6	28.3	0.4
				13.2	91	31.9	1

-*α*^4.2^/ -*α*^4.2^	0	1	0	12.7	92.7	29.9	3

*αα*/ *αα* with HS-40 del	0	0	1	11.2	92.4	29.8	1.1

-* *-^MED-I^ /*αα*	0	0	1	12.1	88.5	29.5	5.2

Hb: hemoglobin, MCV: mean corpuscular volume, MCH: mean corpuscular hemoglobin.

## Data Availability

The datasets analyzed during the current study will be available from the corresponding author on reasonable request.
